# Analysing biodiversity and conservation knowledge products to support regional environmental assessments

**DOI:** 10.1038/sdata.2016.7

**Published:** 2016-02-16

**Authors:** Thomas M. Brooks, H. Resit Akçakaya, Neil D. Burgess, Stuart H.M. Butchart, Craig Hilton-Taylor, Michael Hoffmann, Diego Juffe-Bignoli, Naomi Kingston, Brian MacSharry, Mike Parr, Laurence Perianin, Eugenie C. Regan, Ana S.L. Rodrigues, Carlo Rondinini, Yara Shennan-Farpon, Bruce E. Young

**Affiliations:** 1 International Union for Conservation of Nature, 28 Rue Mauverney, 1196 Gland, Switzerland; 2 World Agroforestry Center (ICRAF), University of the Philippines Los Baños, Laguna 4031, Philippines; 3 School of Geography and Environmental Studies, University of Tasmania, Hobart, Tasmania 7001, Australia; 4 Department of Ecology and Evolution, Stony Brook University, Stony Brook, New York 11794, USA; 5 United Nations Environment Programme World Conservation Monitoring Centre, 219 Huntingdon Road, Cambridge CB3 0DL, UK; 6 Center for Macroecology, Evolution and Climate, Natural History Museum, University of Copenhagen, Copenhagen DK-2100, Denmark; 7 BirdLife International, Wellbrook Court, Cambridge CB2 3QZ, UK; 8 American Bird Conservancy, The Plains, Virginia 20198, USA; 9 The Biodiversity Consultancy, 3E King’s Parade, Cambridge CB1 2RR, UK; 10 Centre d’Ecologie Fonctionnelle et Evolutive, CNRS UMR5175, 1919 Route de Mende, 34293 Montpellier, France; 11 Global Mammal Assessment programme, Department of Biology and Biotechnologies, Sapienza Università di Roma, Viale dell’Università 32, 00185 Roma, Italy; 12 NatureServe, Apdo. 358-1260, Plaza Colonial, San José, Costa Rica

**Keywords:** Biogeography, Conservation biology, Biodiversity, Sustainability

## Abstract

Two processes for regional environmental assessment are currently underway: the Global Environment Outlook (GEO) and Intergovernmental Platform on Biodiversity and Ecosystem Services (IPBES). Both face constraints of data, time, capacity, and resources. To support these assessments, we disaggregate three global knowledge products according to their regions and subregions. These products are: The IUCN Red List of Threatened Species, Key Biodiversity Areas (specifically Important Bird & Biodiversity Areas [IBAs], and Alliance for Zero Extinction [AZE] sites), and Protected Planet. We present fourteen Data citations: numbers of species occurring and percentages threatened; numbers of endemics and percentages threatened; downscaled Red List Indices for mammals, birds, and amphibians; numbers, mean sizes, and percentage coverages of IBAs and AZE sites; percentage coverage of land and sea by protected areas; and trends in percentages of IBAs and AZE sites wholly covered by protected areas. These data will inform the regional/subregional assessment chapters on the status of biodiversity, drivers of its decline, and institutional responses, and greatly facilitate comparability and consistency between the different regional/subregional assessments.

## Background & Summary

Assessment of evidence to underpin responses to challenging societal issues is increasingly recognised as one approach to bridging the science-policy interface. The Intergovernmental Panel on Climate Change is a frequently cited example^1^. This is now paralleled by assessment processes on biodiversity and ecosystem services^2^. However, while much climate change science is primarily global, biodiversity and ecosystem services are much more geographically variable^3^. Their assessment is therefore most useful in a multi-scale framework^4^.

Such multi-scale environmental assessment processes are now underway. The United Nations Environment Programme (UNEP) compiles a periodic GEO. The fifth edition^5^ was published in 2012, and the sixth, now underway, will incorporate regional/subregional assessments. Meanwhile, IPBES has been established, and its regional/subregional assessments are currently beginning^6^.

Global biodiversity and conservation databases hold much relevant information for these assessment processes, given the importance of consistency between different regions. Such databases are typically delivered as spatially explicit global knowledge products, and so can be analysed to inform regional/subregional assessment processes. However, both limited GIS capacity and frequently abbreviated timeframes pose severe challenges to the timely preparation of such analyses. Moreover, such regionalisation is sensitive to assumptions in data preparation and analytical settings, which if not standardised among regions can yield non-comparable results. Given the intention that the GEO and IPBES regional/subregional assessments will feed into respective global assessments, this standardisation is critically important.

Here, as a contribution towards these regional/subregional environmental assessment processes, we provide disaggregations of three scientifically robust and commonly used global knowledge products. These present assessment of the risk of species extinction and associated distributional and other information (The IUCN Red List of Threatened Species), of sites contributing significantly to the global persistence of biodiversity (Key Biodiversity Areas, specifically IBAs and AZE sites), and of protected areas (Protected Planet). We regionalise policy-relevant combinations of each according to the GEO and IPBES regions/subregions. These will inform the regional assessment chapters relating to the status of biodiversity, drivers of its decline, and institutional responses, reflected as, e.g., Chapters 3, 4, and 6 respectively in the IPBES ‘Generic scoping report for the regional and subregional assessments of biodiversity and ecosystem services’ (Decision IPBES-3/1: Work programme for the period 2014–2018, Annex III), and Section 2 ‘State of the Environment’ of the GEO regional assessments in assessing the status and trends of biota and ecosystems. The generic scoping report for IPBES regional/subregional assessments (IPBES/3/6/Add.1), the rationale for which is to promote coherence across the assessments, specifies that these will draw on relevant datasets.

The variation between these regionalisations is remarkable. It provides great support to the regional assessment approach, given that this variation is masked by global reporting. We leave inference regarding causes of this variation to the assessment processes themselves, but note that contributing factors will surely include variation in underlying biogeography, intensity of anthropogenic drivers, and capacity for conservation response.

Disaggregation of other knowledge products would complement our work. Examples could include regionalisation of forest cover^7^, species populations^8^, forest carbon^9^, and protected area management effectiveness, equitability, ecological representativeness, and connectedness^10^. UNEP-WCMC is undertaking such regionalisation for other measures of the state of nature and drivers of change. Other emerging knowledge products, for example to assess risk of ecosystem collapse^11^, and will also be candidates for such disaggregation once they achieve global coverage.

Finally, given the dynamic nature of all three knowledge products used here, we emphasise the importance that assessment processes build capacity for customised analysis. For future regional/subregional analyses, it will be necessary to repeat such analyses, because the data will be outdated by then. If systematic disaggregations of global datasets such as those presented here become unnecessary in the future, because capacity to undertake such analyses has by that point been developed within each region, this would be an excellent measure of the success of the capacity-building efforts associated with processes to assess biodiversity and ecosystem services. Clear guidelines will still be necessary to standardise data analysis across regions, as a precondition for comparisons in space and time.

## Methods

**Figure 1:**
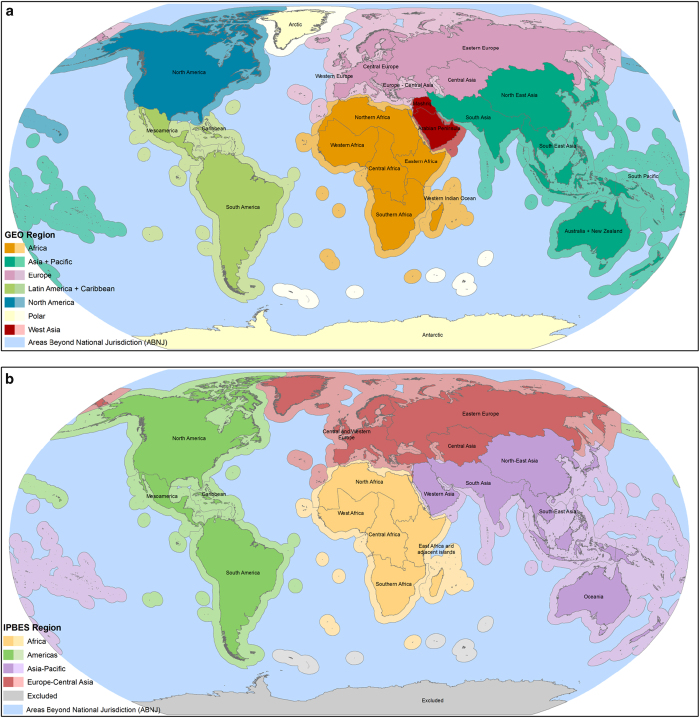
GEO and IPBES regions and subregions. Regionalisation documenting each GEO region and subregion (**a**) and IPBES region and subregion (**b**).

The regionalisations slated for use by GEO^12^ (updated to classify the five Central Asian countries into the ‘Europe’ region) and IPBES^13^ (specifically IPBES-3/1 Annexes IV–VII) are documented in Table 1 (available online only) and shown in Fig. 1. Where data are available, we also include ‘Areas Beyond National Jurisdiction’ (ABNJ) as a region for both GEO and IPBES, because while the high seas are not the subject of regional assessment in these current processes, they may be incorporated into GEO and IPBES in the future. We also include a region for ‘Excluded’ for IPBES, for completeness (this is the Antarctic, which might be included in a future regional IPBES marine assessment). Importantly, these regions do not have (and do not claim to have) any biogeographic basis. Rather they are established based on assumed policy relevance, given economic, cultural, and political similarities among their constituent countries. The three knowledge products that we disaggregate regionally and subregionally comprise assessments of species, important sites, and protected areas. All literature references were accessed 1 September 2015.

### The IUCN Red List of Threatened Species

The IUCN Red List of Threatened Species^14^ (http://www.iucnredlist.org) is a knowledge product derived from assessment of species extinction risk against the IUCN Red List Categories and Criteria^15^. The IUCN Red List of Threatened Species dates back five decades^16^. Stimulated by the 1984 Road to Extinction Conference^17^, a process was initiated to establish quantitative categories and criteria for assessment of extinction risk^18^, and this standard was eventually approved in 2000 by IUCN Council^19^. The nine mutually-exclusive categories of extinction risk are: Not Evaluated (NE); Data Deficient (DD); Least Concern (LC); Near Threatened (NT); Vulnerable (VU); Endangered (EN); Critically Endangered (CR); Extinct in the Wild (EW); and Extinct (EX). In addition, a flag can be applied to denote Critically Endangered species which are ‘Possibly Extinct’ and ‘Possibly Extinct in the Wild’^20^. It incorporates robust protocols for handling uncertainty^21^ and guidelines for application at national and regional levels^22^. Required documentation for all assessments includes not only application of the categories and criteria, but also distribution maps, and application of standard classification schemes, e.g., for threats^23^. Its application is supported by detailed, regularly updated guidelines^24^. As a risk assessment protocol, The IUCN Red List of Threatened Species does not drive priorities for any particular type of action^25^ but rather informs a broad scope of policy and practice ranging from threatened species legislation through to Environmental Impact Assessment^26,27^.

The IUCN Red List of Threatened Species is a dynamic knowledge product. Version 2015-2 includes assessments of 77,340 species against the IUCN Red List Categories and Criteria^19^. These data are maintained in an underlying database, the Species Information Service (SIS), and are freely available for non-commercial use according to published terms^28^, and under data licence for commercial use through IBAT^29^.

All bird species have been assessed six times by BirdLife International^30^, and there have been two assessments of all mammals^31^, amphibians^32^, and reef-building corals^33^. Third reassessments are underway for mammals and amphibians, and comprehensive global assessments of reptiles and fishes are far-advanced, with the former building off existing assessments of all chameleons^14^, and all seasnakes^34^, and the latter all sharks and rays^35^, tarpons and ladyfishes^36^, parrotfishes and surgeonfishes^37^, groupers^38^, tunas and billfishes^39^, and hagfishes^40^, as well as angelfishes, blennies, butterflyfishes, picarels, porgies, pufferfishes, seabreams, sturgeon, and wrasses^14^. Other animal groups that are already comprehensively assessed include freshwater caridean shrimps^41^, cone snails^42^, freshwater crabs^43^, freshwater crayfish^44^, and lobsters^14^. Among plants, comprehensive assessments are complete for cacti^45^, conifers^46^, cycads^47^, seagrasses^48^, and species occurring in mangrove ecosystems^49^. In addition, a sampled approach to Red Listing^50^ has been implemented for reptiles^51^ and dragonflies and damselflies^52^, and is being implemented for various other invertebrate^53^ and plant^54,55^ taxa. The IUCN Red List has a target of assessing 160,000 species, stratified taxonomically, to serve as a ‘barometer of life’ representative across species and ecosystems^56^.

The IUCN Red List of Threatened Species is governed by a Red List Committee, the Chair of which is appointed by the Chair of the IUCN Species Survival Commission (SSC; a position elected by the IUCN Membership of 216 governments and state agencies and 1,043 NGOs at the World Conservation Congress, once every four years). The Red List Committee comprises equal representation from SSC, the Global Species Programme of the IUCN Secretariat, and the Red List Partnership. The latter comprises a dozen institutions who contribute $200,000 or more annually towards the delivery of the Red List Strategic Plan^57^. A Standards & Petitions Sub-Committee, independently appointed by and accountable to the SSC Chair, serves to adjudicate petitions against particular assessments or disputes related to the Red List Categories and Criteria.

**Figure 2:**
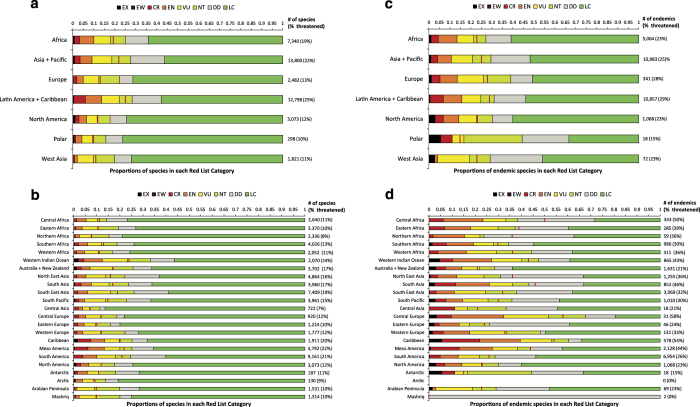
Red List Categories of species occurring in and endemic to each GEO region and subregion. Proportion of species, by Red List Category, in comprehensively assessed groups on The IUCN Red List of Threatened Species (Version 2015-2) occurring in each GEO region (**a**) and subregion (**b**); and proportion of endemic species, by Red List Category, in comprehensively assessed groups on The IUCN Red List of Threatened Species (Version 2015-2) occurring in each GEO region (**c**) and subregion (**d**). The vertical red lines show the best estimate for the proportion of extant species considered threatened (CR, EN and VU) if Data Deficient species are Threatened in the same proportion as data-sufficient species. The numbers to the right of each bar represent the total number of species assessed and in parentheses the best estimate of the percentage threatened. CR, critically endangered; DD, data deficient; EN, endangered; EW, extinct in the wild; EX, extinct; LC, least concern; NT, near threatened; VU, vulnerable.

**Figure 3:**
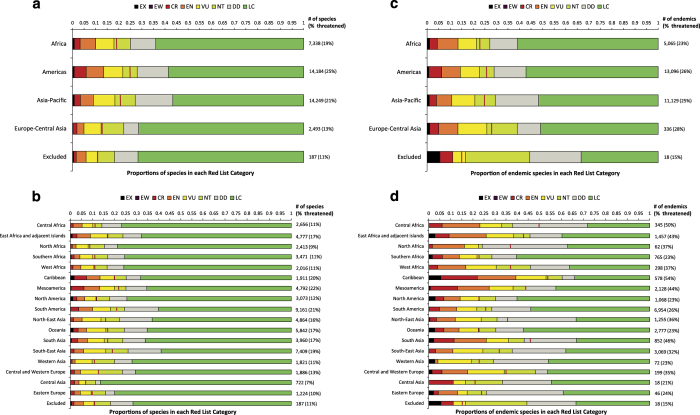
Red List Categories of species occurring in and endemic to each IPBES region and subregion. Proportion of species, by Red List Category, in comprehensively assessed groups on The IUCN Red List of Threatened Species (Version 2015-2) occurring in each IPBES region (**a**) and subregion (**b**); and proportion of endemic species, by Red List Category, in comprehensively assessed groups on The IUCN Red List of Threatened Species (Version 2015-2) occurring in each IPBES region (**c**) and subregion (**d**). The vertical red lines show the best estimate for the proportion of extant species considered threatened (CR, EN and VU) if Data Deficient species are Threatened in the same proportion as data-sufficient species. The numbers to the right of each bar represent the total number of species assessed and in parentheses the best estimate of the percentage threatened. CR, critically endangered; DD, data deficient; EN, endangered; EW, extinct in the wild; EX, extinct; LC, least concern; NT, near threatened; VU, vulnerable.

For larger taxonomic groups that have been assessed comprehensively (i.e., for which >90% of species have been assessed), listed above, we present total numbers of species occurring in each region/subregion, for GEO (Data citation 1, Fig. 2a,b) and IPBES (Data citation 2, Fig. 3a,b). The numbers of species endemic to each region and subregion are shown separately for GEO (Data citation 3, Fig. 2c,d) and IPBES (Data citation 4, Fig. 3c,d). Extinction risk has been assessed for all species in these taxonomic groups. We therefore present in each of the Data citations 1-4 the numbers of species in each Red List Category and the overall percentage threatened (presented as lower, upper and best estimates).

For the taxonomic groups that have been assessed multiple times, it is possible to derive Red List Indices, which are indicators of the aggregate rate at which all species in a given taxonomic group are moving towards extinction^58,59^. Critically, the derivation of the Red List Indices requires extracting only those changes in Red List category between assessments that are caused by genuine increases or decreases in extinction risk, while those caused by changing knowledge or revised taxonomy are accounted for so that they do not drive trends in the index^60^. This avoids the fundamental flaw in earlier approaches to developing indicators from Red Lists^61,62^. The Red List Index has been widely applied as a biodiversity indicator^63–68^. Red List Indices can also be disaggregated by themes including, among others, biogeographic realm, ecosystem, taxonomy, habitat association, threats, ecosystem services, and life-history traits^58,69–75^. For example, the 2010 Millennium Development Goals Report^66^ disaggregated the Red List Index between developed and developing countries (page 57).

It is possible to downscale Red List Indices spatially by combining information on species’ changes in Red List status with the range maps compiled as required documentation for each species’ assessment. For any given taxon and over a given assessment period, the weighted annual change in Red List status is a measure of the relative annual contribution of each region/subregion to the overall change in the global Red List Index for that taxon. Specifically, this measure is calculated for any given region/subregion as a weighted species richness divided by the number of years in the assessment period, where each species is weighted by: i) the number of genuine category changes in The IUCN Red List of Threatened Species during the assessment period (one category change of increasing extinction risk=−1; one category change of decreasing extinction risk=+1), and ii) by the fraction of the species’ range occurring within the region/subregion^76^. Rodrigues *et al.*^76^ successfully applied this downscaling technique to grid cells, ecological regions, and countries. We extend this downscaling to the regionalisation slated for GEO (Data citation 5) and IPBES (Data citation 6).

### Key Biodiversity Areas (specifically IBAs and AZE sites)

Key Biodiversity Areas (KBAs) are sites contributing significantly to the global persistence of biodiversity^77^. They are identified by assessment of sites against standard criteria for the presence of threshold levels of significance for threatened biodiversity (based on Red Lists), range-restricted biodiversity, ecological integrity, and biological process. In 2004, IUCN’s Membership requested ‘a worldwide consultative process to agree a methodology to enable countries to identify Key Biodiversity Areas, drawing on data from the IUCN Red List of Threatened Species and other datasets, building on existing approaches’ (WCC-2004-Res-013). An initial formulation of the scientific basis for identification of KBAs was published by Eken *et al.*^78^, extended into best practice guidelines by Langhammer *et al.*^79^ In response to published critique^80,81^, the World Commission on Protected Areas (WCPA) and SSC convened a joint taskforce to consolidate standards for KBA identification. Building from six technical workshops and 12 regional workshops, this taskforce released a consultation document for public comment^77^ in 2014 and final revisions to this are currently underway for presentation to IUCN’s Council.

The mandate that the process for consolidation of the standard for identification of KBAs must ‘building on existing approaches’ is important, because a number of such processes have been in place for four decades. BirdLife International (then the International Council for Bird Preservation) first established such criteria for the identification of Important Bird Areas (now Important Bird & Biodiversity Areas; IBAs) in the late 1970s. National processes, led by BirdLife International partner NGOs, have now undertaken site assessment following these criteria in >200 countries and territories, yielding identification of >12,800 IBAs in total^30^. The criteria have also been applied in the marine environment to identify >3,000 marine IBAs including 120 in Areas Beyond National Jurisdiction^82^. IBA data are freely available for non-commercial use according to published terms^83^, and under data licence for commercial use through IBAT^29^.

Numerous other organisations have utilised similar criteria to identify important sites for, e.g., amphibians^84^, butterflies^85^, plants^86^, freshwater biodiversity^87^, and marine turtles^88^, mammals^89^, and other biodiversity^90^. In North America, the Natural Heritage Programs have since the 1970s utilised similar criteria to identify ‘B-ranked’ sites^91^. The Alliance for Zero Extinction (AZE), established in 2004 and comprising 88 biodiversity conservation NGOs, is dedicated to the identification and safeguard of all KBAs holding effectively the entire global population of at least one Critically Endangered or Endangered species^92^. A total of 587 AZE sites have been identified, with these data freely available for non-commercial use^93^, and available under data licence for commercial use through IBAT^29^. Among other contributors to the identification of KBAs^94^, the Critical Ecosystem Partnership Fund, which has based its ‘ecosystem profiles’ on KBAs for more than a decade, particularly stands out.

While all of these approaches, and thus KBAs as an umbrella standard, identify sites of importance for biodiversity, these are not necessarily important for any particular type of conservation action. Thus, they are intended to inform, but not prescribe site-level actions for practice and policy, including the establishment of protected areas at national level, and the designation of sites according to regional directives (e.g., Natura 2000 in the European Union) and international conventions (e.g., through the Ramsar Convention on Wetlands of International Importance, natural sites under the World Heritage Convention, Ecologically and Biologically Sensitive Areas in the marine realm under the CBD, etc).

While KBAs have not yet been identified globally using the criteria in the standard being developed, the mandate that the KBA standard and its application must be implemented ‘building on existing approaches’ allows us to have confidence that sites identified by those existing approaches which have been applied globally will be retained as KBAs of international importance under the new standard. The two such existing approaches that have been applied globally are IBAs and AZE sites^95^. Here, we therefore report the total numbers, mean sizes, and percentage coverages of IBAs and AZE sites across the regionalisation proposed for GEO (Data citation 7) and IPBES (Data citation 8).

### Protected Planet

Protected Planet is a knowledge product reporting on the location, status, and management of the world’s protected areas, underpinned by the World Database on Protected Areas^96^ (http://www.protectedplanet.net). It is based on a United Nations Economic and Social Council (ECOSOC) mandate dating back to 1959 for the compilation of the UN List of Protected Areas (ECOSOC Resolution 713 (XXVIII)), and implemented by IUCN-WCPA and the UNEP-World Conservation Monitoring Centre^97^. Protected Planet data are freely available for non-commercial use according to published terms^98^, and under data licence for commercial use through IBAT^29^. It follows the IUCN definition of a protected area as ‘A clearly defined geographical space, recognised, dedicated and managed, through legal or other effective means, to achieve the long-term conservation of nature with associated ecosystem services and cultural values’ across six protected area management categories^99^, where the data are available. Since 1981 it has been maintained as a database, and for the last decade it has been made available as an online knowledge product^100^. In addition to formal, government-reported data, Protected Planet also compiles protected area data from other sources, and is in the process of strengthening its coverage across protected area governance types^101^. In the future, the WDPA is likely to be expanded to include data on ‘other effective area-based conservation measures’, once the definition of such sites has been agreed^97^. This definition is still under discussion^102^, and an IUCN-WCPA task force has been created to provide recommendations on such definition.

Global summary statistics for the coverage of land and sea by different protected area categories are presented in the Protected Planet reports (Fig. 2.8 in Juffe-Bignoli *et al.*^10^). Here, we disaggregate the latest such statistics according to the regionalisations for GEO (Data citation 9), and IPBES (Data citation 10).

An oft-cited limitation of percentage protected area coverage and growth is that it does not account for the distribution of biodiversity which stands to benefit from site-level conservation^103^. Complementing percentage area coverage, much more relevant statistics can be derived by considering the coverage by protected areas of IBAs and AZE sites (Fig. 2c in Butchart *et al.*^104^). These indicators were incorporated into recent assessments of progress against the targets of the Convention on Biological Diversity’s Strategic Plan for Biodiversity^64,68^. Here, we therefore derive the latest regional statistics for: proportions of IBAs fully covered by protected areas, for GEO (Data citation 11, Supplementary Fig. 1) and IPBES (Data citation 12, Supplementary Fig. 2); and proportions of AZE sites fully covered by protected areas, for GEO (Data citation 13, Supplementary Fig. 3) and IPBES (Data citation 14, Supplementary Fig. 4).

## Data Records

### Regional and subregional species diversity and endemism

For both GEO and IPBES, total numbers of species occurring within each region/subregion, and number of species endemic to each region/subregion, are derived using the drop-down menus for countries of occurrence in the SIS, the database which underlies The IUCN Red List of Threatened Species (which are based on documented occurrence in each country), rather than through GIS analysis of the range maps. The only exception is for ABNJ, within which species occurrences are by definition not coded for national occurrence, and so for which we derive occurrence from GIS analysis of species range maps (none of the species included occur only in ABNJ). We count species as occurring if they occur in at least one of a region’s countries; and species as endemic if they are not listed as occurring in any countries outside of a given region (excluding records of vagrants, records of uncertain origin, and introduced populations). We present these data for each of the following taxa: mammals; birds; seasnakes; chameleons; amphibians; sharks and rays; selected bony fish groups (angelfishes; butterflyfishes; tarpons and ladyfishes; parrotfishes and surgeonfishes; groupers; wrasses; tunas and billfishes; hagfishes; sturgeon; blennies; pufferfishes; seabreams; porgies; and picarels); freshwater caridean shrimps; cone snails; freshwater crabs; freshwater crayfish; lobsters; reef-building corals; cacti; conifers; cycads; seagrasses; and plant species occurring in mangrove ecosystems. Resulting data can be found in Data citation 1 and Data citation 3 for GEO (Fig. 2) and in Data citation 2 and Data citation 4 for IPBES (Fig. 3).

### Regional and subregional species prevalence of species extinction risk

For both GEO and IPBES, total numbers of threatened species occurring in and endemic to each region/subregion, and proportions of the total number of species and number of endemic species occurring within each region/subregion that are threatened, are derived from the documented occurrence of species in countries. The only exception is for ABNJ, within which species occurrences are by definition not coded for national occurrence, and so for which we derive occurrence from GIS analysis of species range maps (none of the species included occur only in ABNJ). We count species as occurring if they occur in at least one of a region’s countries; and species as endemic if they are not listed as occurring in any countries outside of a given region (excluding records of vagrants, records of uncertain origin, and introduced populations). We present these data for each of the following taxa: mammals; birds; seasnakes; chameleons; amphibians; sharks and rays; selected bony fish groups (angelfishes and butterflyfishes; tarpons and ladyfishes; parrotfishes and surgeonfishes; groupers; wrasses; tunas and billfishes; hagfishes; sturgeon; blennies; pufferfishes; seabreams; porgies; and picarels); freshwater caridean shrimps; cone snails; freshwater crabs; freshwater crayfish; lobsters; reef-building corals; cacti; conifers; cycads; seagrasses; and plant species occurring in mangrove ecosystems.

The proportion of threatened species can be calculated for all groups that have been comprehensively assessed, but the number of threatened species is often uncertain because it is not known whether Data Deficient (DD) species are actually threatened or not. Some taxonomic groups are much better known than others (i.e., they have fewer DD species), and therefore a more accurate figure for proportion of threatened species can be calculated. Other, less well known groups have a large proportion of DD species, which brings uncertainty into the estimate for proportion of threatened species. The reported percentage of threatened species for each region and sub-region is therefore presented as a best estimate within a range of possible values bounded by lower and upper estimates:

- Lower estimate (‘lower bound’)=% threatened extant species, assuming all DD species are not threatened, i.e., (CR+EN+VU)/(total assessed−EX)
- Best estimate (‘mid-point’)=% threatened extant species, assuming DD species are equally threatened as data sufficient species, i.e., (CR+EN+VU)/(total assessed−EX−DD)
- Upper estimate (‘upper bound’)=% threatened extant species, assuming all DD species are threatened, i.e., (CR+EN+VU+DD)/(total assessed−EX)

Resulting data can be found in Data citation 1 and Data citation 3 for GEO (Fig. 2) and in Data citation 2 and Data citation 4 for IPBES (Fig. 3).

### Regional and Subregional Red List Indices

For both GEO and IPBES, downscaled Red List Indices for mammals, birds, and amphibians are derived as the sum, for all species occurring in the region/subregion, of each species’ number of genuine category changes on The IUCN Red List of Threatened Species (one category change of increasing extinction risk=−1; one category change of decreasing extinction risk=+1), multiplied by the proportion of the species range occurring within the region/subregion, divided by the number of years of the total assessment period^76^. Category changes encompass the following categories, in order of increasing extinction risk: LC; NT; VU; EN; CR; EW and EX (including CR (Possibly Extinct) and CR (Possibly Extinct in the Wild)). Resulting data can be found in Data citation 5 for GEO and in Data citation 6 for IPBES. This approach requires an assumption that changes in extinction risk are evenly spread across all species ranges. While this will never be wholly valid, for regions as broad as those proposed for the three assessment processes in question, it will be a close approximation, because such a high proportion of species are endemic to single regions (86% for GEO, 90% for IPBES) and even to single subregions (66% for both GEO and IPBES).

### Regional and subregional coverage of Key Biodiversity Areas (specifically of IBAs and AZE sites)

For both GEO and IPBES regions and subregions, we present the total numbers and mean sizes of IBAs and AZE sites, and merge site coverage into two layers (one each for IBAs and AZE sites) to calculate percentage areas of each region and subregion covered by IBAs and AZE sites respectively. Resulting data can be found in Data citation 7 for GEO and in Data citation 8 for IPBES.

### Regional and subregional coverage of protected areas

For both GEO and IPBES, we present regional and subregional summary statistics for the coverage of land and sea by different protected area categories. Resulting data can be found in Data citation 9 for GEO and in Data citation 10 for IPBES.

### Regional and subregional trends in coverage of Key Biodiversity Areas (specifically of IBAs and AZE sites) by protected areas

For both GEO and IPBES, we present regional and subregional summary statistics for trends in the proportions of IBAs and of AZE sites fully covered by protected areas, using data on the year of protected area establishment recorded in the January 2013 version of the World Database on Protected Areas. We overlaid digital boundaries of protected areas onto IBAs and AZEs to quantify the degree of overlap. Uncertainty in tracking changes in protected area coverage is generated by the fact that dates of establishment are not documented for 14.3% of terrestrial and 8.6% of marine protected areas. We reflected this by assigning dates of establishment 1,000 times selected at random from those for dated protected areas in the same country to these un-dated sites (or, for countries with less than five protected areas with known year of establishment, from all terrestrial or marine protected areas), and plotting the 95% confidence intervals around median protected area coverage accordingly^64,95,104^. Resulting data can be found in Data citation 11 for GEO for IBAs (Supplementary Fig. 1), in Data citation 13 for GEO for AZE (Supplementary Fig. 3), in Data citation 12 for IPBES for IBAs (Supplementary Fig. 2), and in Data citation 14 for IPBES for AZE (Supplementary Fig. 4). No AZE sites have yet been identified in the GEO Polar region or subregions, the GEO and IPBES West Asia region and subregions, or in the GEO and IPBES Central Asia subregions, and so these are excluded from Data citation 13 and Data citation 14, and from Supplementary Figs 3 and 4, accordingly. No IBAs or AZE sites are covered by protected areas in ABNJ, and so ABNJ are also excluded from Data citation 11, Data citation 12, Data citation 13, Data citation 14 and Supplementary Fig. 1.

## Technical Validation

### The IUCN Red List of Threatened Species

The primary technical validation of the IUCN Red List Categories and Criteria undertaken to date compared actual movement of threatened species through the Red List Categories with that predicted assuming equivalence of the E Criterion (formal quantitative analysis of extinction risk, e.g., using Population Viability Analysis^105^) to the other four criteria^106^. They examined all bird species globally for 1988–2004 and all Australian species for the much longer period, 1750–2000. They found that the expected rates associated with the thresholds for extinction risk used under the E Criterion were broadly consistent with the observed rates, with the exception of category change from Critically Endangered to Extinct, for which the data revealed many fewer extinctions than predicted from the E Criterion thresholds. They speculated that this mismatch is explained by the disproportionate focus of conservation action on Critically Endangered species, and indeed there is good evidence that conservation efforts have prevented the extinction of a substantial number of Critically Endangered species^107,108^. Other approaches to validating the IUCN Red List Categories and Criteria involved retrospective testing based on a reconstructed past extinction^109^, and prospective testing based on projected future extinctions due to climate change^110,111^. Both types of studies concluded that the IUCN criteria can not only identify species that would be extinct without conservation effort, but do so with substantial warning time.

In addition, a number of studies have conducted inter-model comparisons between the IUCN Red List Categories and Criteria and other protocols for assessment of extinction risk^112–114^, comparisons of the consistency of assessments within each of these^115^, and comparisons of the results of application of the IUCN Red List Categories and Criteria with other methods for predicting extinctions such as species-area curves^116^ and random forest decision trees of traits^117^. These found relatively high levels of agreement between the protocols.

Finally, estimates of uncertainty around measurement of threat prevalence (Data citation 1Data citation 2, Data citation 3, Data citation 4, Figs 2 and 3) can be derived from consideration of the numbers of species classified in the Data Deficient category, wherein a species is assessed as having insufficient data available to apply the IUCN Red List Criteria^64^.

### Key Biodiversity Areas (specifically IBAs and AZE sites)

Di Marco *et al.*^118^ undertook a major validation exercise for KBAs, specifically for IBAs in Australia, Europe, and Southern Africa, the three regions from which major independent datasets are available in the form of comprehensive bird atlases. The study set a quantitative conservation targets for all birds in the regions (in terms of number of atlas grid cells to be protected, and compared the selection frequency of atlas grid cells covering IBAs, selected through application of a simulated annealing software (Marxan^119^), with the selection frequency of cells outside IBAs. It found the former to be much higher than the latter, as predicted if such threshold approaches to identification of important sites genuinely identify sites of high irreplaceability for the global persistence of biodiversity. Montesino Pouzols *et al.*^120^ undertook a similar validation, comparing KBAs identified in Madagascar, Myanmar, and the Philippines to results of their global analysis of important areas for terrestrial vertebrates, finding similarly consistent results.

Several tests have also been undertaken to examine the degree to which IBAs represent important sites for biodiversity more generally^121–123^, finding that representation of threatened species in other taxonomic groups is high. Equivalent tests have not yet been undertaken for marine or freshwater biodiversity. Finally, provision also exists for documentation of KBAs as ‘candidate sites’ suspected by experts as holding threshold levels of threatened species, but for taxa that have not yet been formally assessed for the global IUCN Red List or which have not yet been formally documented to occur at the site. No aggregated analyses have yet been undertaken of these candidate sites.

### Protected Planet

The compilation of the World Database on Protected Areas is underpinned by a formal ECOSOC mandate for national submission of protected area datasets to compile the UN List of Protected Areas (ECOSOC Resolution 713 (XXVIII)), technical validation of the data focuses on tracking changes in the completeness of the dataset and working closely with data providers to ensure the veracity and quality of the information provided through a clearly predefined working protocol^97^. The data are collected directly from governmental agencies while data gaps are provided by other authoritative sources such as NGOs or secretariats of international conventions (e.g., Ramsar Convention) or regional entities (e.g., European Environment Agency). All data providers are requested to sign a Data Contributor Agreement which ensures the data published in the WDPA have been approved by the data provider and comply with the terms and conditions of use of the original data.

The quality of the WDPA is ensured by the WDPA data standards^97^ implemented in 2010. As a result, the WDPA has experienced a great improvement in quality in the past years. For example, the proportion of protected areas represented in the dataset as mapped polygons, as opposed to latitude-longitude centre points alone. Between 2003 and 2015, this proportion increased from 39.5 to 91.1%.

## Usage Notes

For the numbers of species and of endemic species in each of the GEO and IPBES regions and sub-regions (Data citations 1–4), we recommend presentation following the horizontal proportional bar chart used in Figs 2 and 3. For relative annual contribution to the global Red List Index (Data citation 5, Data citation 6), coverage of KBAs, specifically IBAs and AZEs (Data citation 7, Data citation 8), and protected area coverage (Data citation 9, Data citation 10), the most appropriate presentation would be in tables, maybe supplemented with absolute bar chart. Changing protected area coverage of KBAs, specifically IBAs and AZEs, over time (Data citations 11–14) is best presented as line graphs, following those presented in Supplementary Fig. 1. In each case, it would be suitable to structure presentation for the regional assessments to show the component subregions. By extension, for the global assessments, it would be suitable to structure them to show the component regions. Table 2 documents the individual datasets published in the data citations.

## Additional Information

Table 1 is only available in the online version of this paper.

**How to cite this article:** Brooks, T. M. *et al.* Analysing biodiversity and conservation knowledge products to support regional environmental assessments. *Sci. Data* 3:160007 doi: 10.1038/sdata.2016.7 (2016).

## Supplementary Material

Supplementary Information

## Figures and Tables

**Table 1:** Regionalisations according to GEO (http://geodata.grid.unep.ch/extras/geo_breakdown.doc) and IPBES (IPBES-3/1, Annexes IV-VII); names strictly follow these two documents, and only countries listed in both are included in the table

Country (GEO)	Country (IPBES)	ISO3	GEO region	GEO sub-region	IPBES region	IPBES sub-region
Afghanistan	Afghanistan	AFG	Asia+Pacific	South Asia	Asia-Pacific	South Asia
Albania	Albania	ALB	Europe	Central Europe	Europe-Central Asia	Central and Western Europe
Algeria	Algeria	DZA	Africa	Northern Africa	Africa	North Africa
American Samoa	American Samoa	ASM	Asia+Pacific	South Pacific	Asia-Pacific	Oceania
Andorra	Andorra	AND	Europe	Western Europe	Europe-Central Asia	Central and Western Europe
Angola	Angola	AGO	Africa	Southern Africa	Africa	Southern Africa
Antigua and Barbuda	Antigua and Barbuda	ATG	Latin America+Caribbean	Caribbean	Americas	Caribbean
Argentina	Argentina	ARG	Latin America+Caribbean	South America	Americas	South America
Armenia	Armenia	ARM	Europe	Eastern Europe	Europe-Central Asia	Eastern Europe
Australia	Australia	AUS	Asia+Pacific	Australia+New Zealand	Asia-Pacific	Oceania
Austria	Austria	AUT	Europe	Western Europe	Europe-Central Asia	Central and Western Europe
Azerbaijan	Azerbaijan	AZE	Europe	Eastern Europe	Europe-Central Asia	Eastern Europe
Bahamas	Bahamas	BHS	Latin America+Caribbean	Caribbean	Americas	Caribbean
Bahrain	Bahrain	BHR	West Asia	Arabian Peninsula	Asia-Pacific	Western Asia
Bangladesh	Bangladesh	BGD	Asia+Pacific	South Asia	Asia-Pacific	South Asia
Barbados	Barbados	BRB	Latin America+Caribbean	Caribbean	Americas	Caribbean
Belarus	Belarus	BLR	Europe	Eastern Europe	Europe-Central Asia	Eastern Europe
Belgium	Belgium	BEL	Europe	Western Europe	Europe-Central Asia	Central and Western Europe
Belize	Belize	BLZ	Latin America+Caribbean	Meso America	Americas	Mesoamerica
Benin	Benin	BEN	Africa	Western Africa	Africa	West Africa
Bhutan	Bhutan	BTN	Asia+Pacific	South Asia	Asia-Pacific	South Asia
Bolivia	Bolivia (Plurinational State of)	BOL	Latin America+Caribbean	South America	Americas	South America
Bosnia and Herzegovina	Bosnia and Herzegovina	BIH	Europe	Central Europe	Europe-Central Asia	Central and Western Europe
Botswana	Botswana	BWA	Africa	Southern Africa	Africa	Southern Africa
Brazil	Brazil	BRA	Latin America+Caribbean	South America	Americas	South America
Brunei Darussalam	Brunei Darussalam	BRN	Asia+Pacific	South East Asia	Asia-Pacific	South-East Asia
Bulgaria	Bulgaria	BGR	Europe	Central Europe	Europe-Central Asia	Central and Western Europe
Burkina Faso	Burkina Faso	BFA	Africa	Western Africa	Africa	West Africa
Burundi	Burundi	BDI	Africa	Eastern Africa	Africa	Central Africa
Cambodia	Cambodia	KHM	Asia+Pacific	South East Asia	Asia-Pacific	South-East Asia
Cameroon	Cameroon	CMR	Africa	Central Africa	Africa	Central Africa
Canada	Canada	CAN	North America	North America	Americas	North America
Cape Verde	Cape Verde	CPV	Africa	Western Africa	Africa	West Africa
Central African Republic	Central African Republic	CAF	Africa	Central Africa	Africa	Central Africa
Chad	Chad	TCD	Africa	Central Africa	Africa	Central Africa
Chile	Chile	CHL	Latin America+Caribbean	South America	Americas	South America
China	China	CHN	Asia+Pacific	North East Asia	Asia-Pacific	North-East Asia
Colombia	Colombia	COL	Latin America+Caribbean	South America	Americas	South America
Comoros	Comoros	COM	Africa	Western Indian Ocean	Africa	East Africa and adjacent islands
Congo	Congo	COG	Africa	Central Africa	Africa	Central Africa
Cook Islands	Cook Islands	COK	Asia+Pacific	South Pacific	Asia-Pacific	Oceania
Costa Rica	Costa Rica	CRI	Latin America+Caribbean	Meso America	Americas	Mesoamerica
Cote d’Ivoire	Cote d’Ivoire	CIV	Africa	Western Africa	Africa	West Africa
Croatia	Croatia	HRV	Europe	Central Europe	Europe-Central Asia	Central and Western Europe
Cuba	Cuba	CUB	Latin America+Caribbean	Caribbean	Americas	Caribbean
Cyprus	Cyprus	CYP	Europe	Central Europe	Europe-Central Asia	Central and Western Europe
Czech Republic	Czech Republic	CZE	Europe	Central Europe	Europe-Central Asia	Central and Western Europe
Democratic People’s Republic of Korea	Democratic People’s Republic of Korea	PRK	Asia+Pacific	North East Asia	Asia-Pacific	North-East Asia
Democratic Republic of the Congo	Democratic Republic of the Congo	COD	Africa	Central Africa	Africa	Central Africa
Denmark	Denmark	DNK	Europe	Western Europe	Europe-Central Asia	Central and Western Europe
Djibouti	Djibouti	DJI	Africa	Eastern Africa	Africa	East Africa and adjacent islands
Dominica	Dominica	DMA	Latin America+Caribbean	Caribbean	Americas	Caribbean
Dominican Republic	Dominican Republic	DOM	Latin America+Caribbean	Caribbean	Americas	Caribbean
Ecuador	Ecuador	ECU	Latin America+Caribbean	South America	Americas	South America
Egypt	Egypt	EGY	Africa	Northern Africa	Africa	North Africa
El Salvador	El Salvador	SLV	Latin America+Caribbean	Meso America	Americas	Mesoamerica
Equatorial Guinea	Equatorial Guinea	GNQ	Africa	Central Africa	Africa	Central Africa
Eritrea	Eritrea	ERI	Africa	Eastern Africa	Africa	East Africa and adjacent islands
Estonia	Estonia	EST	Europe	Central Europe	Europe-Central Asia	Central and Western Europe
Ethiopia	Ethiopia	ETH	Africa	Eastern Africa	Africa	East Africa and adjacent islands
Fiji	Fiji	FJI	Asia+Pacific	South Pacific	Asia-Pacific	Oceania
Finland	Finland	FIN	Europe	Western Europe	Europe-Central Asia	Central and Western Europe
France	France	FRA	Europe	Western Europe	Europe-Central Asia	Central and Western Europe
French Polynesia	French Polynesia	PYF	Asia+Pacific	South Pacific	Asia-Pacific	Oceania
Gabon	Gabon	GAB	Africa	Central Africa	Africa	Central Africa
Gambia	Gambia	GMB	Africa	Western Africa	Africa	West Africa
Georgia	Georgia	GEO	Europe	Eastern Europe	Europe-Central Asia	Eastern Europe
Germany	Germany	DEU	Europe	Western Europe	Europe-Central Asia	Central and Western Europe
Ghana	Ghana	GHA	Africa	Western Africa	Africa	West Africa
Greece	Greece	GRC	Europe	Western Europe	Europe-Central Asia	Central and Western Europe
Grenada	Grenada	GRD	Latin America+Caribbean	Caribbean	Americas	Caribbean
Guam	Guam	GUM	Asia+Pacific	South Pacific	Asia-Pacific	Oceania
Guatemala	Guatemala	GTM	Latin America+Caribbean	Meso America	Americas	Mesoamerica
Guinea	Guinea	GIN	Africa	Western Africa	Africa	West Africa
Guinea-Bissau	Guinea Bissau	GNB	Africa	Western Africa	Africa	West Africa
Guyana	Guyana	GUY	Latin America+Caribbean	South America	Americas	South America
Haiti	Haiti	HTI	Latin America+Caribbean	Caribbean	Americas	Caribbean
Honduras	Honduras	HND	Latin America+Caribbean	Meso America	Americas	Mesoamerica
Hungary	Hungary	HUN	Europe	Central Europe	Europe-Central Asia	Central and Western Europe
Iceland	Iceland	ISL	Europe	Western Europe	Europe-Central Asia	Central and Western Europe
India	India	IND	Asia+Pacific	South Asia	Asia-Pacific	South Asia
Indonesia	Indonesia	IDN	Asia+Pacific	South East Asia	Asia-Pacific	South-East Asia
Iran (Islamic Republic of)	Iran (Islamic Republic of)	IRN	Asia+Pacific	South Asia	Asia-Pacific	South Asia
Iraq	Iraq	IRQ	West Asia	Mashriq	Asia-Pacific	Western Asia
Ireland	Ireland	IRL	Europe	Western Europe	Europe-Central Asia	Central and Western Europe
Israel	Israel	ISR	Europe	Western Europe	Europe-Central Asia	Central and Western Europe
Italy	Italy	ITA	Europe	Western Europe	Europe-Central Asia	Central and Western Europe
Jamaica	Jamaica	JAM	Latin America+Caribbean	Caribbean	Americas	Caribbean
Japan	Japan	JPN	Asia+Pacific	North East Asia	Asia-Pacific	North-East Asia
Jordan	Jordan	JOR	West Asia	Mashriq	Asia-Pacific	Western Asia
Kazakhstan	Kazakhstan	KAZ	Europe	Central Asia	Europe-Central Asia	Central Asia
Kenya	Kenya	KEN	Africa	Eastern Africa	Africa	East Africa and adjacent islands
Kiribati	Kiribati	KIR	Asia+Pacific	South Pacific	Asia-Pacific	Oceania
Kuwait	Kuwait	KWT	West Asia	Arabian Peninsula	Asia-Pacific	Western Asia
Kyrgyzstan	Kyrgyzstan	KGZ	Europe	Central Asia	Europe-Central Asia	Central Asia
Lao People’s Democratic Republic	Lao People’s Democratic Republic	LAO	Asia+Pacific	South East Asia	Asia-Pacific	South-East Asia
Latvia	Latvia	LVA	Europe	Central Europe	Europe-Central Asia	Central and Western Europe
Lebanon	Lebanon	LBN	West Asia	Mashriq	Asia-Pacific	Western Asia
Lesotho	Lesotho	LSO	Africa	Southern Africa	Africa	Southern Africa
Liberia	Liberia	LBR	Africa	Western Africa	Africa	West Africa
Libyan Arab Jamahiriya	Libya	LBY	Africa	Northern Africa	Africa	North Africa
Liechtenstein	Liechtenstein	LIE	Europe	Western Europe	Europe-Central Asia	Central and Western Europe
Lithuania	Lithuania	LTU	Europe	Central Europe	Europe-Central Asia	Central and Western Europe
Luxembourg	Luxembourg	LUX	Europe	Western Europe	Europe-Central Asia	Central and Western Europe
Madagascar	Madagascar	MDG	Africa	Western Indian Ocean	Africa	East Africa and adjacent islands
Malawi	Malawi	MWI	Africa	Southern Africa	Africa	Southern Africa
Malaysia	Malaysia	MYS	Asia+Pacific	South East Asia	Asia-Pacific	South-East Asia
Maldives	Maldives	MDV	Asia+Pacific	South Asia	Asia-Pacific	South Asia
Mali	Mali	MLI	Africa	Western Africa	Africa	West Africa
Malta	Malta	MLT	Europe	Western Europe	Europe-Central Asia	Central and Western Europe
Marshall Islands	Marshall Islands	MHL	Asia+Pacific	South Pacific	Asia-Pacific	Oceania
Mauritania	Mauritania	MRT	Africa	Western Africa	Africa	North Africa
Mauritius	Mauritius	MUS	Africa	Western Indian Ocean	Africa	East Africa and adjacent islands
Mayotte	Mayotte	MYT	Africa	Western Indian Ocean	Africa	East Africa and adjacent islands
Mexico	Mexico	MEX	Latin America+Caribbean	Meso America	Americas	Mesoamerica
Micronesia (Federated States of)	Micronesia (Federated States of)	FSM	Asia+Pacific	South Pacific	Asia-Pacific	Oceania
Moldova, Republic of	Republic of Moldova	MDA	Europe	Eastern Europe	Europe-Central Asia	Eastern Europe
Monaco	Monaco	MCO	Europe	Western Europe	Europe-Central Asia	Central and Western Europe
Mongolia	Mongolia	MNG	Asia+Pacific	North East Asia	Asia-Pacific	North-East Asia
Montenegro	Montenegro	MNE	Europe	Central Europe	Europe-Central Asia	Central and Western Europe
Morocco	Morocco	MAR	Africa	Northern Africa	Africa	North Africa
Mozambique	Mozambique	MOZ	Africa	Southern Africa	Africa	Southern Africa
Myanmar	Myanmar	MMR	Asia+Pacific	South East Asia	Asia-Pacific	South-East Asia
Namibia	Namibia	NAM	Africa	Southern Africa	Africa	Southern Africa
Nauru	Nauru	NRU	Asia+Pacific	South Pacific	Asia-Pacific	Oceania
Nepal	Nepal	NPL	Asia+Pacific	South Asia	Asia-Pacific	South Asia
Netherlands	Netherlands	NLD	Europe	Western Europe	Europe-Central Asia	Central and Western Europe
New Caledonia	New Caledonia	NCL	Asia+Pacific	South Pacific	Asia-Pacific	Oceania
New Zealand	New Zealand	NZL	Asia+Pacific	Australia+New Zealand	Asia-Pacific	Oceania
Nicaragua	Nicaragua	NIC	Latin America+Caribbean	Meso America	Americas	Mesoamerica
Niger	Niger	NER	Africa	Western Africa	Africa	West Africa
Nigeria	Nigeria	NGA	Africa	Western Africa	Africa	West Africa
Niue	Niue	NIU	Asia+Pacific	South Pacific	Asia-Pacific	Oceania
Northern Mariana Islands	Commonwealth of the Northern Mariana Islands	MNP	Asia+Pacific	South Pacific	Asia-Pacific	Oceania
Norway	Norway	NOR	Europe	Western Europe	Europe-Central Asia	Central and Western Europe
Occupied Palestinian Territory	State of Palestine	PSE	West Asia	Mashriq	Asia-Pacific	Western Asia
Oman	Oman	OMN	West Asia	Arabian Peninsula	Asia-Pacific	Western Asia
Pakistan	Pakistan	PAK	Asia+Pacific	South Asia	Asia-Pacific	South Asia
Palau	Palau	PLW	Asia+Pacific	South Pacific	Asia-Pacific	Oceania
Panama	Panama	PAN	Latin America+Caribbean	Meso America	Americas	Mesoamerica
Papua New Guinea	Papua New Guinea	PNG	Asia+Pacific	South Pacific	Asia-Pacific	Oceania
Paraguay	Paraguay	PRY	Latin America+Caribbean	South America	Americas	South America
Peru	Peru	PER	Latin America+Caribbean	South America	Americas	South America
Philippines	Philippines	PHL	Asia+Pacific	South East Asia	Asia-Pacific	South-East Asia
Pitcairn Island	Pitcairn Island	PCN	Asia+Pacific	South Pacific	Asia-Pacific	Oceania
Poland	Poland	POL	Europe	Central Europe	Europe-Central Asia	Central and Western Europe
Portugal	Portugal	PRT	Europe	Western Europe	Europe-Central Asia	Central and Western Europe
Qatar	Qatar	QAT	West Asia	Arabian Peninsula	Asia-Pacific	Western Asia
Republic of Korea	Republic of Korea	KOR	Asia+Pacific	North East Asia	Asia-Pacific	North-East Asia
Reunion	Reunion	REU	Africa	Western Indian Ocean	Africa	East Africa and adjacent islands
Romania	Romania	ROU	Europe	Central Europe	Europe-Central Asia	Central and Western Europe
Russian Federation	Russian Federation	RUS	Europe	Eastern Europe	Europe-Central Asia	Eastern Europe
Rwanda	Rwanda	RWA	Africa	Eastern Africa	Africa	East Africa and adjacent islands
Saint Kitts and Nevis	Saint Kitts and Nevis	KNA	Latin America+Caribbean	Caribbean	Americas	Caribbean
Saint Lucia	Saint Lucia	LCA	Latin America+Caribbean	Caribbean	Americas	Caribbean
Saint Vincent and the Grenadines	Saint Vincent and the Grenadines	VCT	Latin America+Caribbean	Caribbean	Americas	Caribbean
Samoa	Samoa	WSM	Asia+Pacific	South Pacific	Asia-Pacific	Oceania
San Marino	San Marino	SMR	Europe	Western Europe	Europe-Central Asia	Central and Western Europe
Sao Tome and Principe	Sao Tome and Principe	STP	Africa	Central Africa	Africa	Central Africa
Saudi Arabia	Saudi Arabia	SAU	West Asia	Arabian Peninsula	Asia-Pacific	Western Asia
Senegal	Senegal	SEN	Africa	Western Africa	Africa	West Africa
Serbia	Serbia	SRB	Europe	Central Europe	Europe-Central Asia	Central and Western Europe
Seychelles	Seychelles	SYC	Africa	Western Indian Ocean	Africa	East Africa and adjacent islands
Sierra Leone	Sierra Leone	SLE	Africa	Western Africa	Africa	West Africa
Singapore	Singapore	SGP	Asia+Pacific	South East Asia	Asia-Pacific	South-East Asia
Slovakia	Slovakia	SVK	Europe	Central Europe	Europe-Central Asia	Central and Western Europe
Slovenia	Slovenia	SVN	Europe	Central Europe	Europe-Central Asia	Central and Western Europe
Solomon Islands	Solomon Islands	SLB	Asia+Pacific	South Pacific	Asia-Pacific	Oceania
Somalia	Somalia	SOM	Africa	Eastern Africa	Africa	East Africa and adjacent islands
South Africa	South Africa	ZAF	Africa	Southern Africa	Africa	Southern Africa
Spain	Spain	ESP	Europe	Western Europe	Europe-Central Asia	Central and Western Europe
Sri Lanka	Sri Lanka	LKA	Asia+Pacific	South Asia	Asia-Pacific	South Asia
Sudan	Sudan	SDN	Africa	Northern Africa	Africa	North Africa
Suriname	Suriname	SUR	Latin America+Caribbean	South America	Americas	South America
Swaziland	Swaziland	SWZ	Africa	Southern Africa	Africa	Southern Africa
Sweden	Sweden	SWE	Europe	Western Europe	Europe-Central Asia	Central and Western Europe
Switzerland	Switzerland	CHE	Europe	Western Europe	Europe-Central Asia	Central and Western Europe
Syrian Arab Republic	Syrian Arab Republic	SYR	West Asia	Mashriq	Asia-Pacific	Western Asia
Tajikistan	Tajikistan	TJK	Europe	Central Asia	Europe-Central Asia	Central Asia
Thailand	Thailand	THA	Asia+Pacific	South East Asia	Asia-Pacific	South-East Asia
The former Yugoslav Republic of Macedonia	The former Yugoslav Republic of Macedonia	MKD	Europe	Central Europe	Europe-Central Asia	Central and Western Europe
Timor-Leste	Timor-Leste	TLS	Asia+Pacific	South East Asia	Asia-Pacific	South-East Asia
Togo	Togo	TGO	Africa	Western Africa	Africa	West Africa
Tokelau	Tokelau	TKL	Asia+Pacific	South Pacific	Asia-Pacific	Oceania
Tonga	Tonga	TON	Asia+Pacific	South Pacific	Asia-Pacific	Oceania
Trinidad and Tobago	Trinidad and Tobago	TTO	Latin America+Caribbean	Caribbean	Americas	Caribbean
Tunisia	Tunisia	TUN	Africa	Northern Africa	Africa	North Africa
Turkey	Turkey	TUR	Europe	Central Europe	Europe-Central Asia	Central and Western Europe
Turkmenistan	Turkmenistan	TKM	Europe	Central Asia	Europe-Central Asia	Central Asia
Tuvalu	Tuvalu	TUV	Asia+Pacific	South Pacific	Asia-Pacific	Oceania
Uganda	Uganda	UGA	Africa	Eastern Africa	Africa	East Africa and adjacent islands
Ukraine	Ukraine	UKR	Europe	Eastern Europe	Europe-Central Asia	Eastern Europe
United Arab Emirates	United Arab Emirates	ARE	West Asia	Arabian Peninsula	Asia-Pacific	Western Asia
United Kingdom of Great Britain and Northern Ireland	United Kingdom of Great Britain and Northern Ireland	GBR	Europe	Western Europe	Europe-Central Asia	Central and Western Europe
United Republic of Tanzania	United Republic of Tanzania	TZA	Africa	Southern Africa	Africa	East Africa and adjacent islands
United States of America	United States of America	USA	North America	North America	Americas	North America
Uruguay	Uruguay	URY	Latin America+Caribbean	South America	Americas	South America
Uzbekistan	Uzbekistan	UZB	Europe	Central Asia	Europe-Central Asia	Central Asia
Vanuatu	Vanuatu	VUT	Asia+Pacific	South Pacific	Asia-Pacific	Oceania
Venezuela	Venezuela (Bolivarian Republic of)	VEN	Latin America+Caribbean	South America	Americas	South America
Viet Nam	Viet Nam	VNM	Asia+Pacific	South East Asia	Asia-Pacific	South-East Asia
Wallis and Futuna	Wallis and Futuna	WLF	Asia+Pacific	South Pacific	Asia-Pacific	Oceania
Western Sahara	Western Sahara	ESH	Africa	Northern Africa	Africa	North Africa
Yemen	Yemen	YEM	West Asia	Arabian Peninsula	Asia-Pacific	Western Asia
Zambia	Zambia	ZMB	Africa	Southern Africa	Africa	Southern Africa
Zimbabwe	Zimbabwe	ZWE	Africa	Southern Africa	Africa	Southern Africa
Other overseas territories and dependencies are included throughout in the regions to which they are closest geographically (which may differ from the region from which they are governed). GEO also identifies a separate Polar region, encompassing Greenland (included within Europe-Central Asia by IPBES) and Antarctica (not yet proposed for IPBES regional assessment). Shapefiles for both regionalisations are provided (http://dx.doi.org/10.5061/dryad.6gb90.2/15.2), both to document these regionalisations, and to support the authors of the respective GEO and IPBES regional and subregional assessments in standardising further spatial analyses.						

**Table 2:** Individual datasets published in the data citations.

**File name**	**Description**	**Authors**	**Dryad DOI**
Total_Species_GEO.csv	Total numbers of species and of threatened species occurring in each GEO region and subregion	IUCN	http://dx.doi.org/10.5061/dryad.6gb90.2/1.2
Total_Species_IPBES.csv	Total numbers of species and of threatened species occurring in each IPBES region and subregion		http://dx.doi.org/10.5061/dryad.6gb90.2/2.2
Endemic_Species_GEO.csv	Total numbers of species and of threatened species endemic to each GEO region and subregion		http://dx.doi.org/10.5061/dryad.6gb90.2/3.2
Endemic_Species_IPBES.csv	Total numbers of species and of threatened species endemic to each IPBES region and subregion		http://dx.doi.org/10.5061/dryad.6gb90.2/4.2
Red_List_Index_GEO.csv	Relative annual contribution to the global Red List Index for mammals, birds, and amphibians in each GEO region and subregion	IUCN & BirdLife International	http://dx.doi.org/10.5061/dryad.6gb90.2/5.2
Red_List_Index_IPBES.csv	Relative annual contribution to the global Red List Index for mammals, birds, and amphibians in each IPBES region and subregion		http://dx.doi.org/10.5061/dryad.6gb90.2/6.2
IBAs_AZEs_GEO.csv	Total numbers, mean sizes, and percentage coverages of IBAs and AZEs in each GEO region and subregion	BirdLife International & AZE	http://dx.doi.org/10.5061/dryad.6gb90.2/7.2
IBAs_AZEs_IPBES.csv	Total numbers, mean sizes, and percentage coverages of IBAs and AZEs in each IPBES region and subregion		http://dx.doi.org/10.5061/dryad.6gb90.2/8.2
PAs_GEO.csv	Percentage protected area coverage of land and sea, for each GEO region and subregion	UNEP-WCMC & IUCN	http://dx.doi.org/10.5061/dryad.6gb90.2/9.2
PAs_IPBES.csv	Percentage protected area coverage of land and sea, for each IPBES region and subregion		http://dx.doi.org/10.5061/dryad.6gb90.2/10.2
Protected_IBAs_GEO.csv	Percentage of IBAs wholly covered by protected areas, over time, for each GEO region and subregion	BirdLife International, IUCN & UNEP-WCMC	http://dx.doi.org/10.5061/dryad.6gb90.2/11.2
Protected_IBAs_IPBES.csv	Percentage of IBAs wholly covered by protected areas, over time, for each IPBES region and subregion		http://dx.doi.org/10.5061/dryad.6gb90.2/12.2
Protected_AZEs_GEO.csv	Percentage of AZE sites wholly covered by protected areas, over time, for each GEO region and subregion	AZE, BirdLife International, IUCN & UNEP-WCMC	http://dx.doi.org/10.5061/dryad.6gb90.2/13.2
Protected_AZEs_IPBES.csv	Percentage of AZE sites wholly covered by protected areas, over time, for each IPBES region and subregion		http://dx.doi.org/10.5061/dryad.6gb90.2/14.2
