# Supplementary material for: Analysing biodiversity and conservation knowledge products to support regional environmental assessments
Source: Sci Data. 2016 Feb 16;3:160007. doi: 10.1038/sdata.2016.7 (PMC4755129; doi:10.1038/sdata.2016.7)
Supplement: Supplementary Information [file sdata20167-s1.pdf]

Supplemental Figures: Table of Contents

Supplementary Figure 1. Growth in proportion of IBAs fully covered by protected areas for each of the GEO regions and subregions. Each row corresponds to a region, wherein we first present the results for the region and then for each of its subregions. 95% confidence intervals are omitted for clarity but are provided in Data citation 11.

Supplementary Figure 2. Growth in proportion of IBAs fully covered by protected areas for each of the IPBES regions and subregions. Each row corresponds to a region, wherein we first present the results for the region and then for each of its subregions. 95% confidence intervals are omitted for clarity but are provided in Data citation 12.

Supplementary Figure 3. Growth in proportion of AZE sites fully covered by protected areas for each of the GEO regions and subregions. Each row corresponds to a region, wherein we first present the results for the region and then for each of its subregions. “No data” indicates regions/subregions within which no AZE sites have yet been identified. Each row corresponds to a region, wherein we first present the results for the region and then for each of its subregions. 95% confidence intervals are omitted for clarity but are provided in Data citation 13.

Supplementary Figure 4. Growth in proportion of AZE sites fully covered by protected areas for each of the IPBES regions and subregions. Each row corresponds to a region, wherein we first present the results for the region and then for each of its subregions. “No data” indicates regions/subregions within which no AZE sites have yet been identified. Each row corresponds to a region, wherein we first present the results for the region and then for each of its subregions. 95% confidence intervals are omitted for clarity but are provided in Data citation 14.

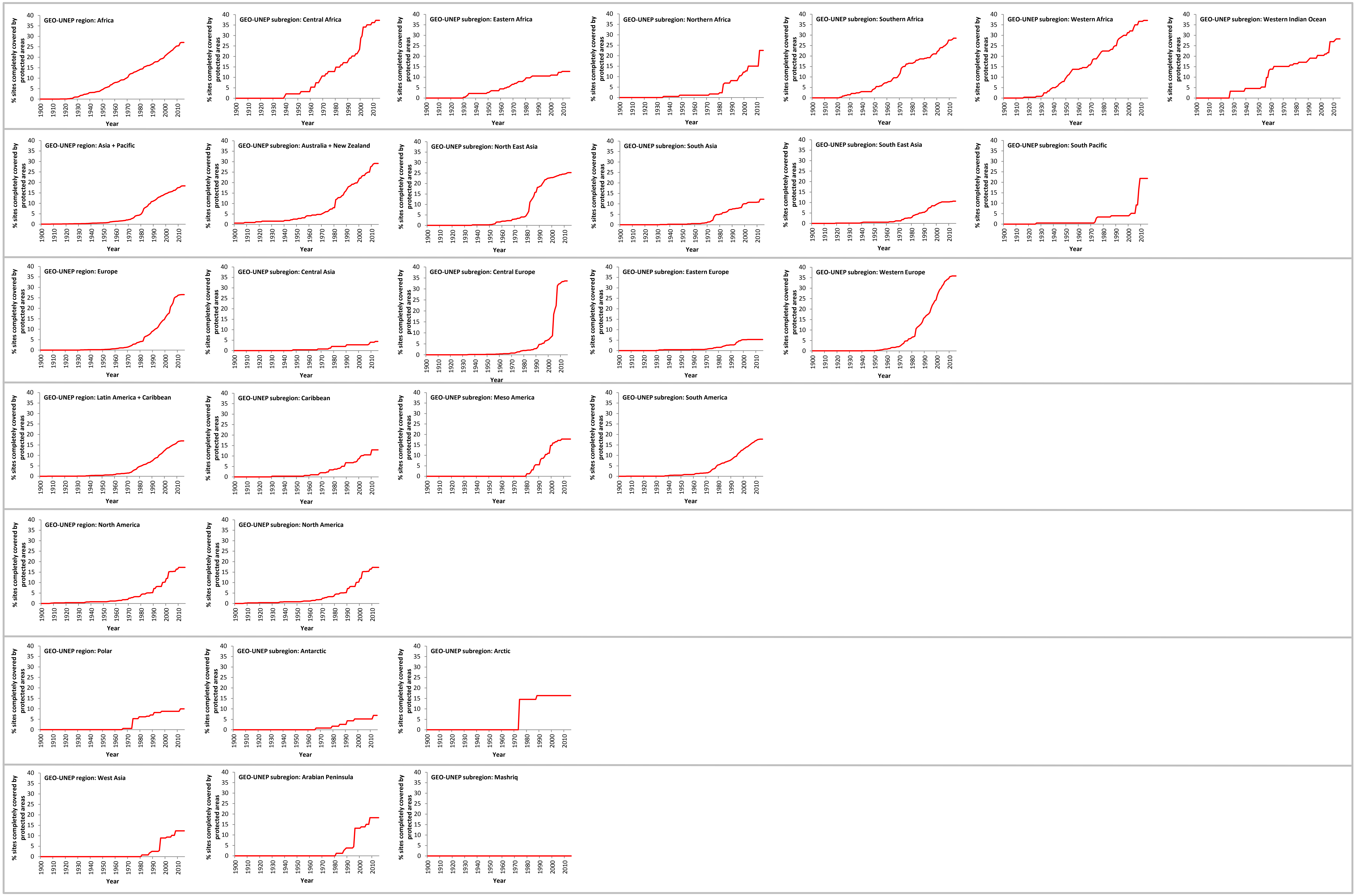

Supplementary Figure 1. Growth in proportion of IBAs fully covered by protected areas for each of the GEO regions and subregions. Each row corresponds to a region, wherein we first present the results for the region and then for each of its subregions. 95% confidence intervals are omitted for clarity but are provided in Data citation 11.

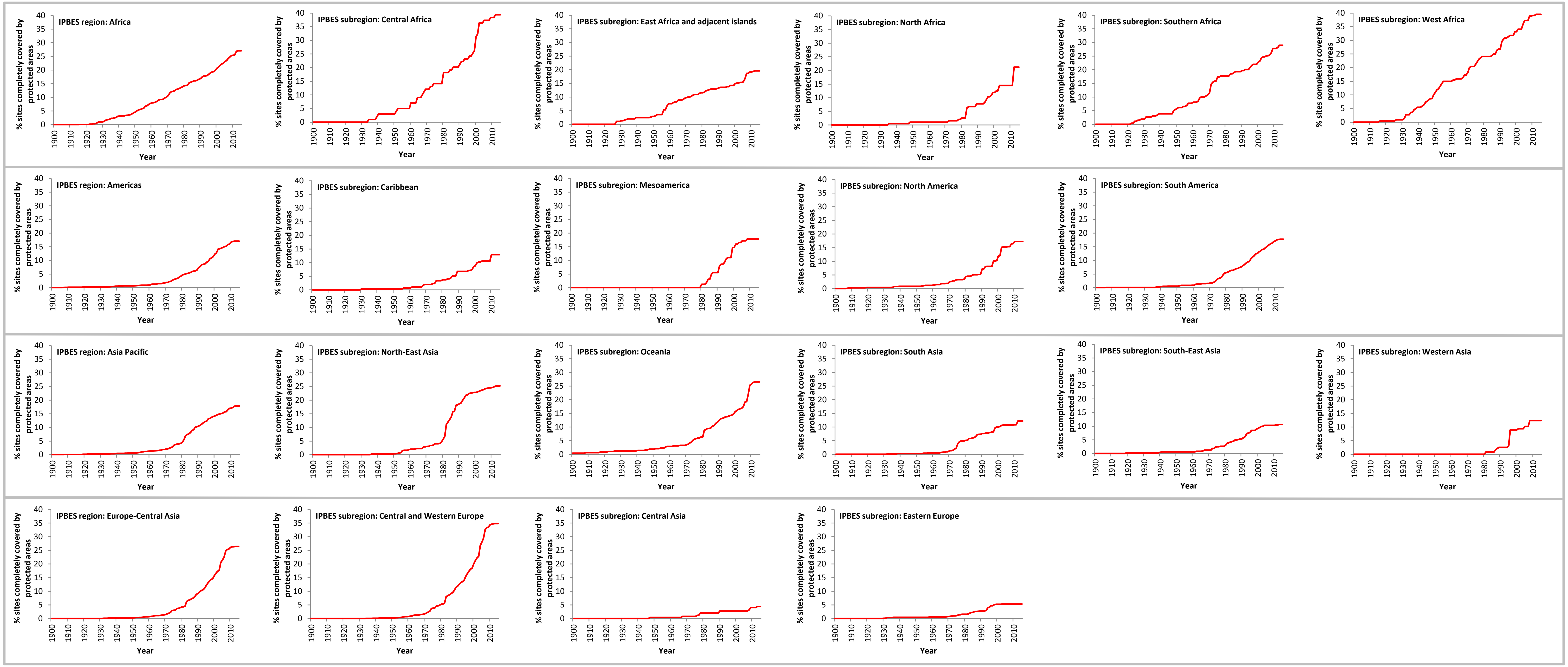

Supplementary Figure 2. Growth in proportion of IBAs fully covered by protected areas for each of the IPBES regions and subregions. Each row corresponds to a region, wherein we first present the results for the region and then for each of its subregions. 95% confidence intervals are omitted for clarity but are provided in Data citation 12.

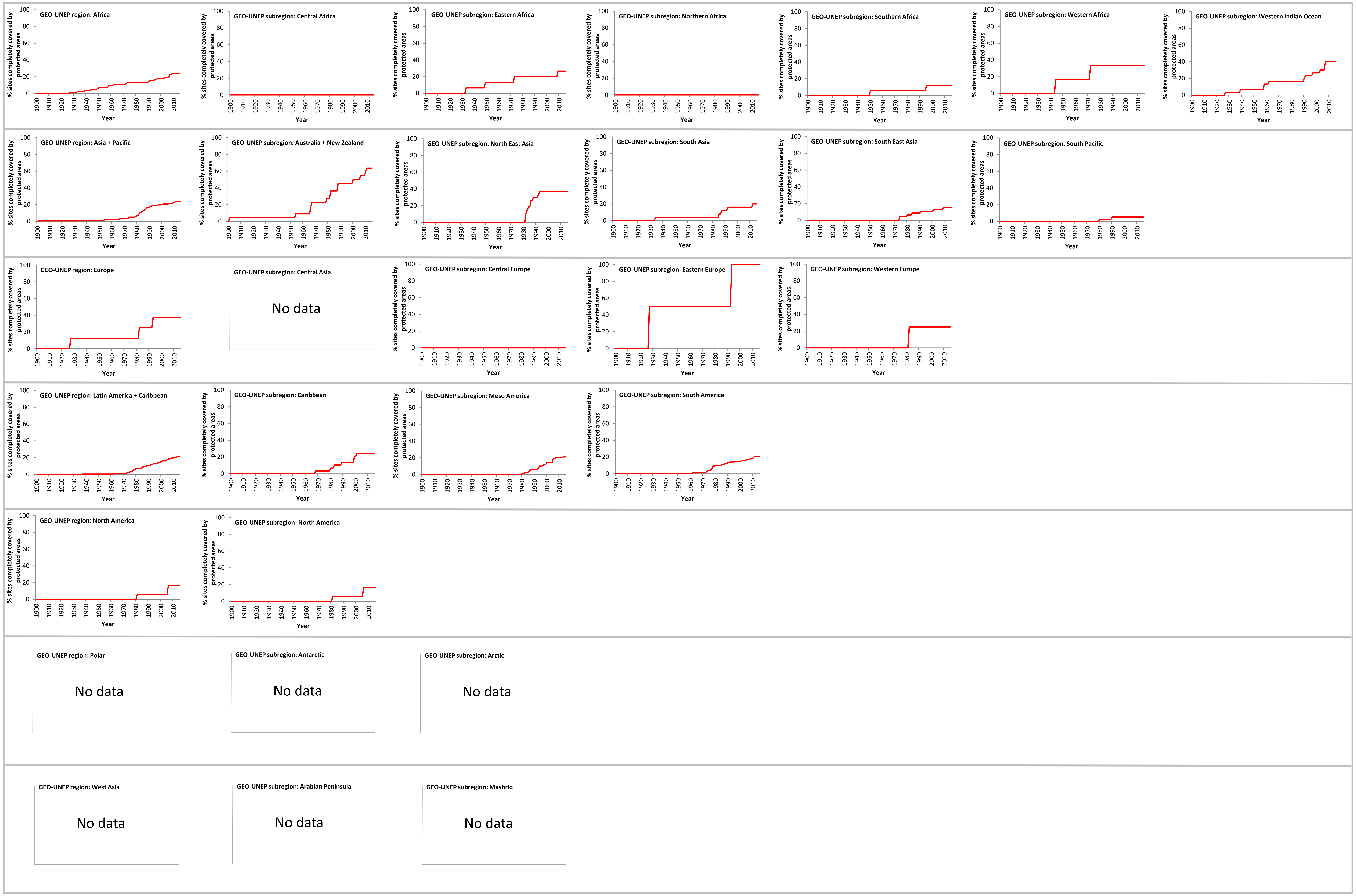

Supplementary Figure 3. Growth in proportion of AZE sites fully covered by protected areas for each of the GEO regions and subregions. Each row corresponds to a region, wherein we first present the results for the region and then for each of its subregions. “No data” indicates regions/subregions within which no AZE sites have yet been identified. Each row corresponds to a region, wherein we first present the results for the region and then for each of its subregions. 95% confidence intervals are omitted for clarity but are provided in Data citation 13.

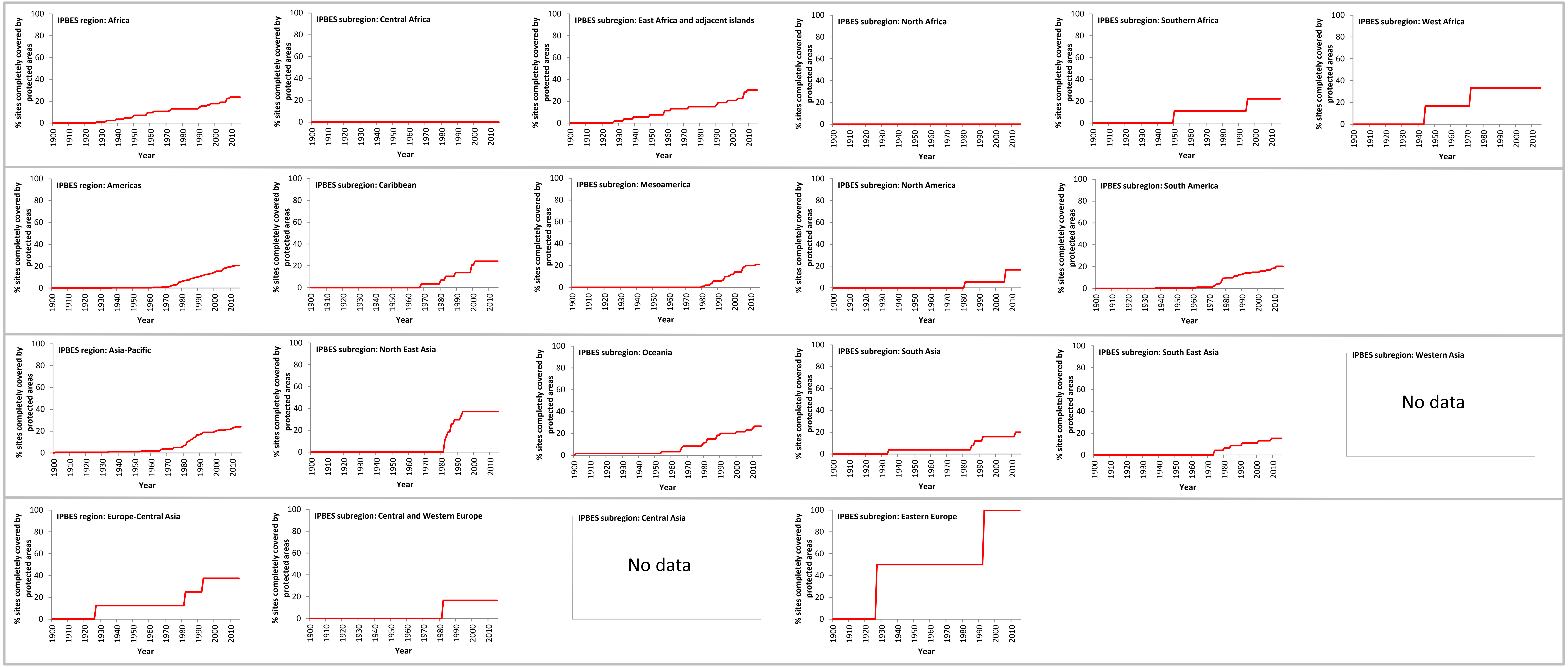

Supplementary Figure 4. Growth in proportion of AZE sites fully covered by protected areas for each of the IPBES regions and subregions. Each row corresponds to a region, wherein we first present the results for the region and then for each of its subregions. “No data” indicates regions/subregions within which no AZE sites have yet been identified. Each row corresponds to a region, wherein we first present the results for the region and then for each of its subregions. 95% confidence intervals are omitted for clarity but are provided in Data citation 14.
